# Quality of life after idiopathic multicentric Castleman disease in China: a cross-sectional, multi-center survey of patient reported outcome and caregiver reported outcome

**DOI:** 10.1186/s13023-024-03450-0

**Published:** 2024-12-19

**Authors:** Jia Chen, Miao-yan Zhang, Yu-han Gao, Lu Zhang, Jian Li

**Affiliations:** 1 Department of Hematology, Peking Union Medical College Hospital, Chinese Academy of Medical Sciences and Peking Union Medical College, Beijing, China; 2 State Key Laboratory of Complex Severe and Rare Diseases, Peking Union Medical College Hospital, Chinese Academy of Medical Sciences and Peking Union Medical College, Beijing, China

**Keywords:** Idiopathic multicentric Castleman disease, Patient reported outcome, Caregiver reported outcome

## Abstract

The majority of multicentric Castleman disease (MCD) patients in China are of the idiopathic subtype (iMCD) with systemic manifestations. However, the impact of iMCD on life quality, mental and psychological status, social function, and caregiving burden is poorly understood. To address this gap, a cross-sectional web-based survey was conducted with 178 iMCD patients and 82 caregivers, including 42 patient-caregiver dyads. Patient-reported outcome measurements were performed using four self-administered questionnaires (MCD-SS, SF-36, PHQ-9, and WPAI:GH). Caregiver-reported outcome measurements were performed using three questionnaires (SF-36, Zarit-22, CRA) to assess the caregiving burden. Correlation analysis was performed in patient-caregiver dyads. Patients reported a median of nine symptoms by MCD-SS, with a high mean score of 4.34 for the Fatigue domain. Their SF-36 scores indicated significant declines in physical and mental health compared to the Chinese general population (*p* < 0.001). Based on PHQ-9, around 65% of patients exhibited depressive symptoms, with 28.6% of them experiencing mild to severe major depression. Only 47.2% (84/178) of the patients were employed, and 28.5% experienced impaired work time. Caregivers also reported lower SF-36 scores than the general population (*p* < 0.05) and expressed feeling of self-criticism, lack of family support and financial problems. Correlations were observed between patients’ symptom burden, mental health impairment and caregiving burden (correlation coefficient: 0.31 ~ 0.55). Our study concluded that fatigue and depressive symptoms significantly impact the life quality and social well-being of iMCD patients. The disease also affect the physical and mental health of caregivers, leading to feelings of guilt and a lack of family support.

**Supplementary Information:**

The online version contains supplementary material available at 10.1186/s13023-024-03450-0.

## Introduction

Castleman Disease (CD) is a rare proliferative disease of the lymph nodes with heterogeneous clinical manifestations. It is divided into Unicentric Castleman disease (UCD) and Multicentric Castleman Disease (MCD) according to the extent of lymph node involvement [1]. MCD can be further categorized into idiopathic (iMCD), HHV-8-positive MCD, and POEMS syndrome-associated MCD. The iMCD patients are further classified into iMCD-TAFRO and iMCD-not otherwise specified (iMCD-NOS) [2]. In China, the majority of MCD patients are iMCD with systemic manifestations, including fever, fatigue, weight loss, itching, rash, systemic lymphadenopathy, hepatosplenomegaly, and inflammatory state [3]. The prognosis of iMCD is relatively poor, and the main treatment goal is to control clinical symptoms and achieve the remission of measurable indices [4–6].

Patient-reported outcome measures (PROMs) are self-reported tools to assess subjective symptom burden and psychological well-being related to health conditions or treatments [7, 8]. PROMs may be designed to evaluate the symptoms or emotional burden of a specific disease or focus on the impact of disease on general perceptions, such as quality of life and social function [9, 10]. In recent years, multiple studies have explored the role of PROMs in oncology patients. PROMs have been utilized in clinical trials as secondary endpoints or to evaluate side effects [11].

Informal caregivers, whether they are a spouse, a family member, or a friend, play a critical role in supporting cancer patients and are crucial to the overall success of treatment, yet there is limited focus on their well-being [12]. The incorporation of primary caregiver outcome measures, termed caregiver-reported outcomes measurements (CROMs) [13, 14], has received little attention. CROMs evaluate the burden of care and the impact of caregiving on the general health of caregivers. Notably, the use of CROMs iMCD has not been explored. Prioritizing the well-being of both patients and their caregivers is essential for achieving optimal treatment outcomes.

Given the potentially high symptom burden in iMCD, accurately and reliably assessing the impact of reported symptoms on the physical and mental health of patients, as well as on their daily life and on the burden of caregiving, is of utmost importance. However, there is currently limited research demonstrating the importance of PROMs in iMCD patients or CROMs in caregivers. To our knowledge, only one validated PROM for MCD symptoms, the MCD-Symptom Scale (MCD-SS) [15], has been used in the assessment of MCD patients in a phase 2 clinical trial of siltuximab [11], and in patient/caregiver survey [16, 17]. A single international online survey, involving 51 iMCD patients and 11 informal caregivers, focused on the disease burden of iMCD reported by patients or by caregivers [17]. The impact of symptom burden and caregiving burden in iMCD has not been adequately captured. This study aims to address this gap through the use of PROMs and CROMs, in order to comprehensively describe the disease burden in iMCD patients and caregivers, focusing on their quality of life, mental and psychological status, and social function.

## Methods

### Study design and setting

A cross-sectional survey was conducted from May 2023 to November 2023 across China, targeting individuals aged 18 and above who had been diagnosed with iMCD. Eligible patients and their caregivers were identified through House of Castleman, which is the first patient-led organization for Castleman disease in China [18, 19]. Patient medical records and clinical and laboratory data were reviewed, and the diagnosis of iMCD was confirmed by their doctors based on the diagnostic criteria proposed by the Castleman Disease Collaborative Network (CDCN) [1, 18]. Exclusion criteria included individuals under the age of 18, those unable to complete an online questionnaire, or with a history of mental illness. The survey was administered online, coordinated by researchers. In patients, four self-administered questionnaires were used to comprehensively assess symptoms, quality of life, physical and mental health, and social function. In caregivers, three questionnaires were performed to assess the caregiving burden on family and caregivers' life quality. All PROMs and CROMs were collected via WeChat. Patients' clinical characteristics were obtained from their questionnaire responses, and their treatment status was extracted from physician records and clinical data collected during consultations [18]. Caregivers' demographic information were also collected in the questionnaire. Patient-caregiver dyads were identified using patients' provided name, gender, and age.

Following the ethical standards of the 1964 Declaration of Helsinki and its later amendments, this study was reviewed and approved by the Ethics Committee of Peking Union Medical College Hospital. All study participants were informed that participation in the study was voluntary. Informed consent for online survey participation and clinical data extraction was obtained via signed electronic consent forms. Participants consented by returning a completed questionnaire or declined by not responding, or by returning an unanswered survey.

### Measurements in iMCD patients and caregivers

The MCD-SS is a 16-item questionnaire developed to assess a patient’s perception of MCD-related symptom severity [15]. It includes items related to Fatigue (tiredness, fatigue, lack of energy, feeling weak), Rash/Itching (sores/rash on skin, itch), Sweats (night sweats, daytime sweating), and other symptoms (cough, shortness of breath, fever, loss of appetite, numbness or tingling, pain, swollen lymph nodes, swelling or edema). Patients were asked to recall their symptoms over the previous 24 h and rate their severity on a 6-point scale from very mild (score = 1) to very severe (score = 5), with an option to indicate they did not experience the symptom (score = 0). The domain scores were rescaled to a range of 0–10, with higher scores indicating more severe symptoms. The MCD-SS total score was derived by combining the scores from the Fatigue, Rash/Itching, and Sweats domains, as well as seven of the eight items that were not included in a specific domain. Fever was excluded from the total score calculations because it was deemed more appropriate to assess it using actual temperature data rather than relying on patient reports. Therefore, the total score denominator was set at ten to encompass 16 items.

The Short Form (SF)−36 is a validated 36-item self-reported measure used in both cancer patients and the general population [20]. It includes eight domains: physical functioning (PF), role-physical (RP), bodily pain (BP), general health (GH), vitality (VT), social functioning (SF), role-emotional (RE) and mental health (MH). Scores range from 0 to 100, with higher scores representing a higher perceived health. In our study, SF-36 was utilized to assess the life quality of both iMCD patients and their caregivers.

PHQ-9 is a self-administered nine-item instrument to assess depressive symptoms based on the DSM-IV (Diagnostic and Statistical Manual of Mental Disorders, Fourth Edition) classification for major depressive disorder [21]. Each item is scored on a scale of 0–3, resulting in an overall score ranging from 0 to 27. Interpretation of PHQ-9 scores is as follows: no depression (score 0–5), mild depression (score 5–9), moderate depression (score 10–14), moderately severe depression (score 15–19), severe depression (score 20–27). Previous studies have demonstrated that a PHQ-9 score ≥ 10 had a sensitivity of 83–92% and a specificity of 82–88% for diagnosing major depression, using mental health professional re-interviews as the criterion standard [21, 22]. Additionally, literature supports the reliability and validity of the PHQ-9 in assessing depressive symptoms among Chinese patients [23]. In our study, we used a PHQ-9 score of ≥ 10 as the threshold for diagnosing clinical depression in patients with iMCD.

The Work Productivity and Activity Impairment Questionnaire: General Health (WPAI:GH) is an online instrument comprising six items designed to estimate absenteeism and presenteeism in the general population [24]. The WPAI:GH assesses four outcomes: absenteeism, presenteeism, overall work impairment, and activity impairment. To aid interpretation, the scores for these outcomes are converted into percentages by multiplying them by 100, with higher scores indicating greater impairment or lower productivity. It is worth noting that the Chinese version of the WPAI:GH has been validated in previous studies, supporting its reliability and validity for use in the Chinese population [25].

The Caregiver Reaction Assessment (CRA) is a 24-item instrument designed to assess multidimensional caregiving experiences [26]. The items are subscaled into five dimensions: impact on schedule, financial problems, lack of family support, health problems, and positive experiences of caregiving. Each item is rated on a 5-point scale from strongly disagree (score = 1) to strongly agree (score = 5) [27]. The reliability and validity of the Chinese version of CRA have been evaluated in cancer patients’ caregivers [28, 29].

The Zarit burden interview (Zarit-22) is a 22-item caregiver-reported psychometric questionnaire designed to measure the burden experienced by the respondent in providing care to the patient [30]. The items assess the perceived impact of caregiving on the caregiver’s physical health, emotional health, social activities, and financial situation. Each item is answered on a 5-point scale, with higher scores indicating higher perceived burden. The Zarit-22 has been tested in samples of caregivers of adult patients with physical and mental illness, as well as in palliative care settings [31, 32]. Literature has supported the reliability and validity of the Zarit-22 in assessing caregiving burden among caregivers in China [33].

### Statistical analysis

Patient clinical and demographic characteristics were reported using descriptive statistics. Student’s t-test or the Mann–Whitney U test was used for continuous outcomes. Reported outcomes in paired samples were compared by paired sample Wilcoxon test. Categorical data were analyzed with the chi-square test or Fisher’s exact test. Pearson or Spearman’s correlation analysis was applied to measure the monotonic relationship between reported outcomes for parametric or non-parametric test, respectively. Tukey’s method was utilized in multiple comparisons. Factors with a *p* < 0.05 were believed to be highly clinically significant and independent predictors. Statistical analyses were performed using R software (version 3.6.3), SPSS (version 25, IBM Corp., Armonk, NY), and GraphPad Prism (version 10.01). All tests were two-tailed, and *p* < 0.05 was considered statistically significant.

## Results

### Survey responses

**Fig. 1:**
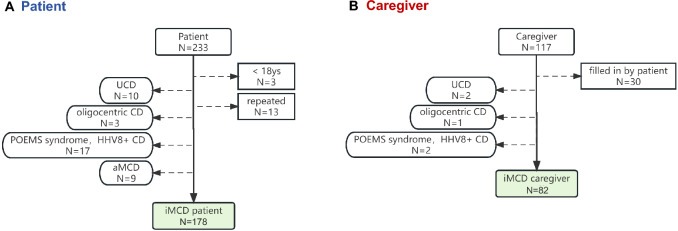
iMCD patients and caregivers included in the study. **A** 178 of 233 patients met the inclusion criteria and completed the online survey. **B** 82 of 177 caregivers met the inclusion criteria and completed the online survey. CD: Castleman Disease, UCD: unicentric CD; aMCD: asymptomatic multicentric CD; iMCD: idiopathic multicentric CD; ys: years old

The survey took approximately 7–15 min to complete. For patient reported outcomes, a total of 233 patients consented and completed the online survey. Among them, 178 (76.4%) were identified as having iMCD and met the inclusion criteria (Fig. 1). Reasons for exclusion included a diagnosis other than iMCD, age younger than 18 years, or multiple survey submissions. Patients were from various regions across China (Fig. S1). For caregiver reported outcome, 117 caregivers consented and completed the online survey. Eighty-two (70.1%) of them met the inclusion criteria, with reasons for exclusion including the diagnosis of other subtypes of MCD or completion of the questionnaire by the patient (Fig. 1). The demographic data of the patients and caregivers are presented in Table 1.

**Table 1:** Demographic characteristics of iMCD patients and caregivers

Characteristics of iMCD patients	N = 178
Age, median (range)	43 (18–73)
*Gender, N (%)*	
Male	95 (53.3%)
Female	83 (46.6%)
*Sub type, N(%)*	
iMCD-TAFRO	11 (6.2%)
iMCD-NOS	167 (93.8%)
Duration from diagnosis, months	16 (1–280)
*Treatment, N (%)*	
untreated	29 (16.3%)
in first-line	95 (53.4%)
in second-line	20 (11.2%)
≥ third line	18 (10.1%)
in remission	16 (9.0%)
*Education, N (%)*	
Primary education	13 (7.3%)
Lower secondary education	35 (19.7%)
Upper secondary education	45 (25.2%)
Advanced education	81 (45.5%)
Unkown	4 (2.2%)

Characteristics of iMCD caregivers	N = 82
Age, median (range)	42 (21–73)
*Gender, N (%)*	
Male	32 (39.0%)
Female	50 (61.0%)
*Relationship, N (%)*	
Spouse	39 (47.6%)
Parents	19 (23.2%)
Children	21 (25.6%)
Siblings	3 (3.7%)
*Education, N (%)*	
Primary education	2 (2.4%)
Lower secondary education	18 (22.0%)
Upper secondary education	19 (23.2%)
Advanced education	41 (50.0%)
Unkown	2 (2.4%)
*Past medical history, N (%)*	
Healthy	65 (79.3%)
*Unhealthy	17 (20.7%)
*Duration of caregiving, N (%)*	
Less than 3 months	18 (22.0%)
3–6 months	13 (15.9%)
7–12 months	4 (4.9%)
More than 1 year	47 (57.3%)
*Frequency of caregiving, N (%)*	
Everyday	45 (54.9%)
More than 4 days a week	6 (7.3%)
1–3 days a week	8 (9.8%)
Several days a month	7 (8.5%)
Several days a year	9 (11.0%)
Independent	2 (2.4%)
Prefer not to answer	5 (6.1%)

^*^Unhealthy: history of hypertension, coronary disease, gastric ulcers, diabetes, hyperlipidemia, hyperthyroidism, hypothyroidism, turberculosis, skin infection, choleliths

### Patient clinical characteristics

The male-to-female ratio of patients was 1.14 (Table 1). At the time of the survey, the median age of patients was 43 years, with a small proportion being 65 years or older (3.9%). The median time from diagnosis was 16 months. Eleven of 178 patients were diagnosed with iMCD-TAFRO (6.2%). At the time of the survey, most of the patients (74.7%) were receiving CD-specific treatment, while 16.3% remained untreated, and 9.0% were in remission. The most common treatment regimens included thalidomide-cyclophosphamide-prednisone (TCP regimen) (46/178, 25.8%), siltuximab/tocilizumab (39/178, 21.9%), bortezomib-cyclophosphamide-dexamethasone (BCD regimen) (32/178, 18.0%), and other treatments like combined chemotherapy.

### Patient reported outcomes

#### MCD-SS

**Fig. 2:**
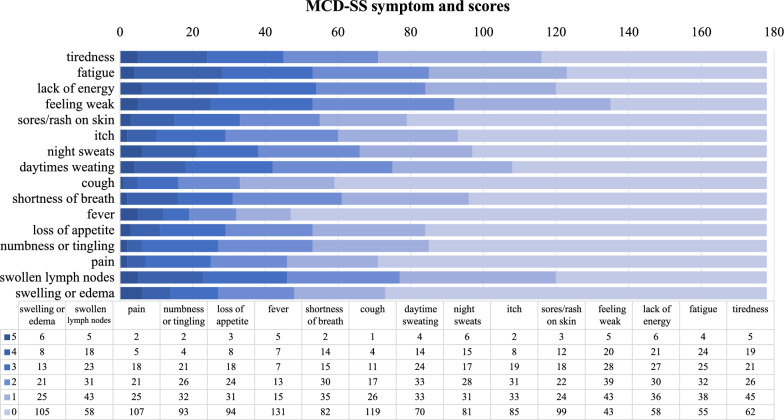
MCD-SS symptoms and scores. Scores of each symptom and the number of patients at every score are displayed. MCD-SS: MCD symptom scale

**Fig. 3:**
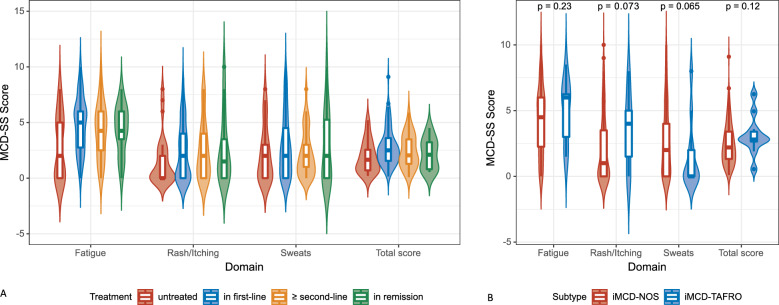
MCD-SS in treatment and disease subgroups of iMCD patients. **A** Scores of each MCD-SS domain grouped by treatment status. **B** Scores of each MCD-SS domain in iMCD-TAFRO and iMCD-NOS. MCD-SS: MCD symptom scale

For MCD-SS, the median number of symptoms reported per subject was nine (range 1–16) out of a total of 16. The mean total score was 2.50 (standard deviation (SD) 1.57), indicating that the overall symptom severity at the time of the survey was generally mild. The mean score was 4.34 (SD 2.56) for the Fatigue domain, 2.28 (SD 2.54) for the Rash/Itching domain, and 2.63 (SD 2.65) for the Sweats domain. Approximately 50% of scores of the question in the Fatigue domain were moderate or higher. Additionally, 51.7% of the patients had moderate to very severe swollen lymph nodes (Fig. 2). In subgroup analysis, patients at different treatment statuses had relatively high scores in the Fatigue domain (Fig. 3A). The total and component scores of MCD-SS were not significantly different between patients in the middle of treatment and in remission. Although the difference was not significant, patients diagnosed with iMCD-TAFRO tend to have higher scores of Rash/Itching (mean score 3.64 vs. 2.19, *p* = 0.075) and lower score of Sweats (mean score 1.55 vs. 2.70, *p* = 0.065) as compared to those in iMCD-NOS (Fig. 3B).

#### SF-36

Table 2 provides a summary of SF-36 results for all dimensions for the iMCD patients surveyed. The iMCD average scores by dimension are compared with caregivers and the Chinese reference values (based on 1688 individuals, with a mean age 46.0 years, and 50.9% male) [34]. Compared to the general population, iMCD patients reported a significant decline in the mean scores of most domains (*p* < 0.001), except for “physical function” and “mental health”. Of the patients treated with systemic therapy for over a year, 45 (45.0%) patients reported their general health status as “good” or “very good” at the time they completed the questionnaire.When asked about changes in health status compared to a year ago, 19 (19.0%) reported a worsening of their health, with 12 (12.0%) rating their health as “somewhat worse now than one year ago” and seven (7.0%) as “much worse now than a year ago”. The remaining participants rated their health as “about the same” (N = 20; 20.0%), “somewhat better now than a year ago” (N = 19; 19.0%) or “much better now than a year ago” (N = 42; 42.0%).

**Table 2:** Component scores (mean, SD) of SF-36 in iMCD patients, caregivers, and the general Chinese population [34]

Domain	Patients	Caregivers	Chinese Population	P1	P2	P3
Physical function	80.4 (18.9)	87.4 (17.9)	82.2 (19.8)	0.021	0.476	0.051
Role-physical	57.0 (44.0)	68.3 (42.5)	81.2 (33.6)	0.042	< 0.001	0.003
Body Pain	74.1 (23.7)	75.2 (21.3)	81.5 (20.5)	0.917	< 0.001	0.006
General Health	48.0 (21.8)	63.8 (21.1)	56.7 (20.2)	< 0.001	< 0.001	0.006
Vitality	59.6 (22.0)	61.2 (22.8)	52.0 (20.9)	0.837	< 0.001	< 0.001
Social-functioning	68.2 (26.2)	75.1 (23.5)	83.0 (17.8)	0.018	< 0.001	0.001
Role-emotional	57.9 (44.4)	64.6 (42.9)	84.4 (32.4)	0.306	< 0.001	< 0.001
Mental health	63.3 (20.3)	60.7 (20.3)	59.7 (22.7)	0.659	0.103	0.918

P1: adjusted *P* value of comparisons between patients and caregivers, P2: adjusted *P* value of comparisons between patients and Chinese population, P3: adjusted *P* value of comparisons between caregivers and Chinese population

*SD* Standard deviation

#### PHQ-9

**Fig. 4:**
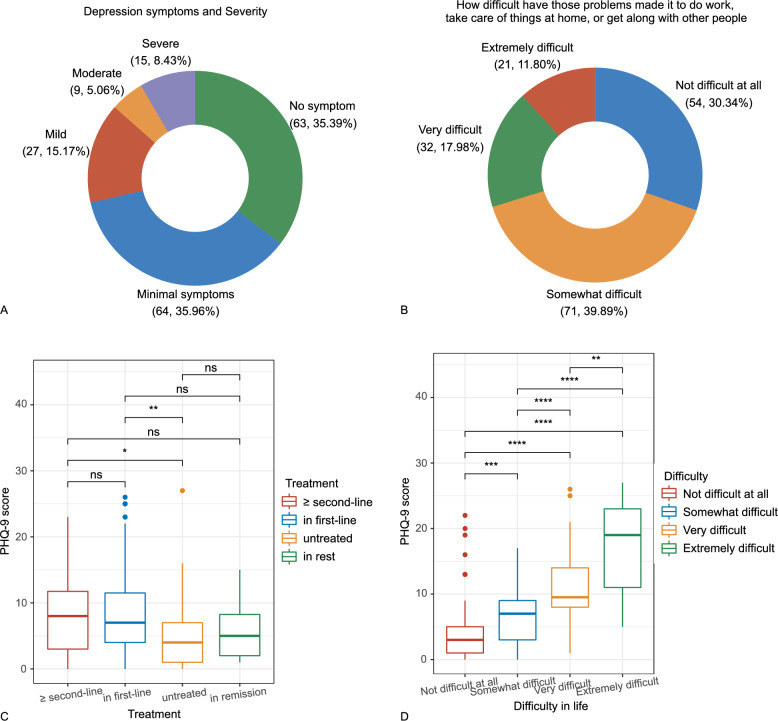
PHQ-9 scores in iMCD patients. **A** Distribution of depressive symptoms and major depression detected by PHQ-9 in iMCD patients (N, %). **B** Degree of difficulties in work, daily lives and social well-being caused by depressive symptoms (N, %). **C** PHQ-9 score of patients in different treatment status. **D** PHQ-9 score of patients with varied degree of difficulty in lives. *P value < 0.05; **P value < 0.005; ***P value < 0.0005; ****P value < 0.00005; *****P value < 0.000005

The mean score of PHQ-9 in iMCD patients was 7.8 (SD 6.4). Based on the PHQ-9 scores, approximately 65% of the patients exhibited depressive symptoms, and 28.6% of the patients might have mild to severe major depression (PHQ-9 score ≥ 10) (Fig. 4A). Furthermore, about 70% of the patients experiencing difficulties related to depression that affected their work, life at home, or relationship with others (Fig. 4B). The degree of difficulty was positively associated with PHQ-9 scores. Additionally, the mean PHQ-9 score of untreated patients was lower than those of patients in the first-line therapy (5.1 vs. 8.8, *P* = 0.001) or second-line therapy (5.1 vs. 8.4, *P* = 0.010), but was not significantly different from that of patients in remission (5.1 vs. 6.0, *P* = 0.260). In our results, PHQ-9 score was not significantly correlated with patients’ age, gender, educational level, or disease subtype.

#### WPAI:GH

In the overall sample of 178 patients, 84 (47.2%) reported that they were currently employed. Being employed was associated with younger age (median age 40 years vs. 46 years, *p* = 0.031), gender (male ratio of 63.1% vs. 44.7%, *p* = 0.014), higher education (56.0% vs. 36.2%, *p* = 0.008), and lower levels of fatigue as measured by MCD-SS (mean score 3.8 vs. 4.8, *p* = 0.008). However, working status was not significantly different between patients diagnosed with iMCD-TAFRO or iMCD-NOS (36.4% vs. 47.9%, p = 0.425), as well as in the presentation of PHQ-9 score and MCD-SS total score (*p* = 0.100 ~ 0.170).

Out of the 84 patients who were employed, 37 (44.0%) reported missing work in the past week due to their health problems, accounting for 28.5% of their working time (absenteeism). Among the 81 patients who worked in the past seven days, 21.2% of their work was impaired due to their health problem (presenteeism). Furthermore, 29.4% of the patients' regular daily activities were hindered due to their health problems (activity impairment) (Table S1, Fig. S2).

#### Correlation analyses

Correlation analyses were conducted among patient-reported outcomes (Fig. S3A). A higher score in the Fatigue domain of the MCD-SS was significantly associated with lower scores of all dimensions of SF-36 outcomes (r: −0.52 ~ −0.39), higher PHQ-9 scores (r: 0.42), and greater work or activity impairment (r: 0.19 ~ 0.43). This suggests that prominent fatigue symptoms in iMCD patients exacerbate their physical and mental health and impaired their ability to work and carry out daily lives. Additionally, a higher PHQ-9 score was significantly associated with worse presentation on the SF-36 (r: 0.49 ~ 0.63) and WPAI:GH (r: 0.56 ~ 0.61), underscoring the importance of paying attention to the depression symptoms in iMCD patients. Furthermore, weak correlations were found between PRO outcomes and patients’ age, gender, duration of disease, and disease subtype (r: −0.26 ~ 0.21).

### Caregiver characteristics

The median age of caregivers was 42 (range: 21–73). The majority of caregivers in our sample were female (61.0%), had received advanced education (50.0%), and reported physical problems (79.3%). About half of the caregivers were the spouse of the patients (47.6%), followed by children (25.6%), parents (23.2%) and siblings (3.7%). More than half of the caregivers had been taking care of the patients for more than one year (57.3%). Everyday care was needed for more than half of the patients (54.9%) (Table 1).

### Caregiver reported outcome

#### SF-36

Compared with the Chinese reference values [34], most SF-36 component scores in iMCD caregivers were higher than those of iMCD patients but lower than those of the general population (Table 2). Taking care of the iMCD patients added physical and mental burden to the caregivers, mostly in the decrease of the mean score of “role-emotional” (64.6 vs. 84.4, *p* < 0.001), “social-functioning” (75.1 vs. 83.0, *p* = 0.001), “body pain” (75.2 vs. 81.5, *p* = 0.006), and “role-physical” (68.3 vs. 81.2, *p* = 0.003) compared to the general Chinese population. In the 82 iMCD caregivers surveyed, 61 of them (74.4%) reported their general health status as “good” or better at the time they completed the questionnaire. Compared with a year ago, 28 caregivers (34.1%) reported a worsening in their health status, with 25 (30.5%) rated their health as “somewhat worse now than one year ago” and three (3.7%) as “much worse now than a year ago”. Caregivers with better education status reported a heavier impact on the SF-36 role-physical domain (r: 0.27) and SF-36 social functioning domain (r: 0.22) (Fig. S3B).

#### Burden of caregiving measured by Zarit-22 and Caregiver Reaction Assessment

The psychological burden and caregiving experience were measured using Zarit-22 and CRA (Table 3). Among all iMCD caregivers surveyed, 20 (24.4%) of them reported “severe” or “moderate to severe” burden, 34 (41.5%) of them reported “mild to moderate” burden, and 28 (34.1%) reported “no to mild” burden. The feeling of self-criticism was prominent in caregivers (mean score 5.1, SD 2.3). Results of the caregivers’ CRA ratings showed relatively high score in the lack of family support (mean score 3.5, SD 0.4) and financial problems (mean score 3.4, SD 0.5). The burden of caregiving was positively correlated with the frequency of care (r: 0.17 ~ 0.34), the age of the caregiver (r: 0.20 ~ 0.29), and male gender (r: 0.16) (Fig. S3B). Caregiver with worse presentations on the SF-36 reported heavier burden of care (Fig. S3B).

**Table 3:** Burden of caregiving measured by Zarit-22 and Caregiver Reaction Assessment

Domain	Mean (SD)	Max. score
*Zarit-22*		
Sacrifice	10.5 (7.0)	32
Loss of control	6.1 (4.1)	16
Embarrassment	3.4 (3.7)	16
Self-criticism	5.1 (2.3)	8
Dependency	2.3 (2.3)	12
Overall burden	1.4 (1.2)	4
Toltal score	28.8 (16.8)	88
*Caregiver reaction assessment*		
Disrupted schedule	3.1 (0.3)	5
Financial problems	3.4 (0.5)	5
Lack of family support	3.5 (0.4)	5
Health problems	3.0 (0.4)	5
Self-esteem	3.0 (0.5)	5

Max score: full mark of the domain

*SD* Standard deviation

### Paired analysis

Forty-two patient-caregiver dyads were recruited for the study. Among them, the ratio of patients diagnosed with iMCD-TAFRO in patient-caregiver dyads was higher than in all individuals surveyed (16.7% vs. 6.2%, *p* = 0.026). Other demographic characteristics of the dyads were not significantly different with the whole group (Table S2).

**Fig. 5:**
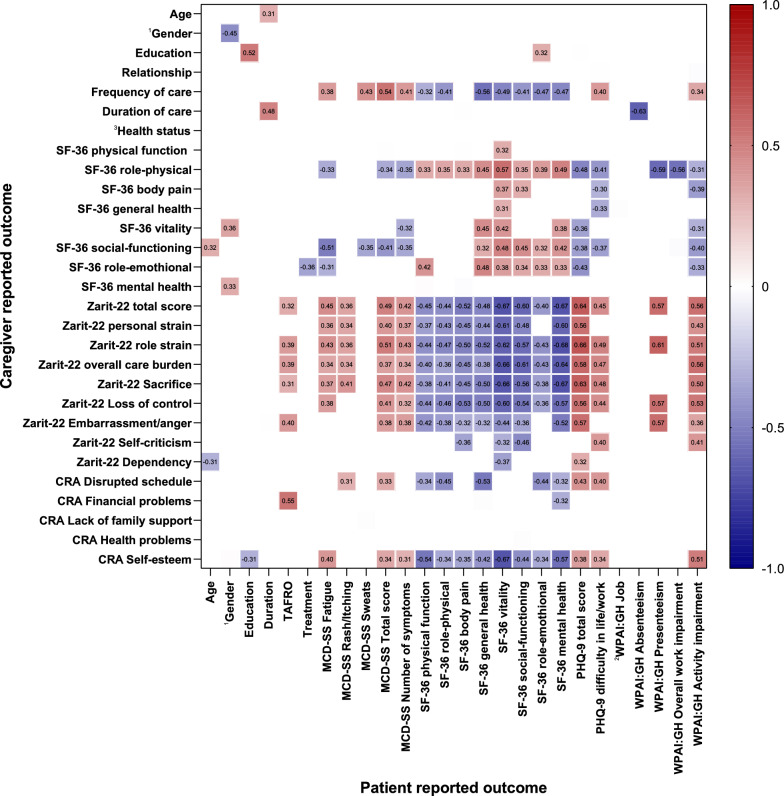
Correlations between patient-reported outcomes and caregiver-reported outcomes in 42 patient-caregiver dyad. Spearman’s rank correlation coefficient, or Spearman rho, is annotated in the figure. Correlations with p value < 0.05 are displayed.1Gender: male = 1, female = 2; ^2^WPAI:GH JOB: able for work = 1, unable for work = 2; ^3^Health status: healthy = 1, unhealthy = 2

Correlations were computed to examine the relationships between patient physical and mental health, social well-being, and caregiver burden (Fig. 5). Caregivers of iMCD-TAFRO patients had higher levels of family financial problems and psychometric burden (r: 0.31 ~ 0.55). The frequency of care was positively associated with the symptom burden measured by MCD-SS (r: 0.38 ~ 0.54), and negatively associated with patients’ quality of life measured by SF-36 (r: −0.56 ~ −0.32). Caregivers who took care of the patients with heavier symptom burden and worse quality of life experienced a heavier caregiving burden. Additionally, caregivers’ quality of life was associated with patients’ MCD symptom burden and depressive symptom burden (r: −0.51 ~ −0.30). Moderate correlations were found between patients’ work and activity impairment and caregiver burden (r: 0.41 ~ 0.61).

## Discussion

Our study is one of the first cross-sectional reports to describe the symptom burden, quality of life, mental and psychological status, social function, and the burden of care of iMCD using PROMs and CROMs. In our results, fatigue and depressive symptoms were prevalent in iMCD patients, affecting the quality of life and social well-being. Most patients reported an improvement in their quality of life after a year of treatment, highlighting the potential impact of interventions on patient outcomes. However, further longitudinal studies are needed to assess the correlations between patient-reported outcomes and the clinical response to treatment. Regarding the burden of care, our study found that the disease may also affect the physical and mental health of iMCD caregivers. Feelings of guilt and lack of family support were reported by iMCD caregivers. Aged and male caregivers exhibited more adverse effects on their health and social lives. The overall burden of care was correlated with iMCD symptom burden, general health and the depressive symptoms of iMCD patients. More attention is required to address the fatigue and depressive symptoms of iMCD patients and to provide support for their caregivers.

A previous international online survey of 51 iMCD patients and 11 caregivers, conducted in North America and European countries, utilized a 45-question instrument developed from the MCD-SS, SF-36, and CarerQol-7D [17]. This survey, refined to reflect the specific disease burden of iMCD, found that patients reported a mean of 6.7 symptoms, most of which had a moderate to severe impact on daily life. Caregivers also experienced significant burdens, including impacts on their social life, freedom, emotional well-being, travel/relocation, and work. Further validation may be needed for this 45-question survey before its use in future studies or clinical practice [35]. In contrast, our study employed a generally accepted online survey without refinement. Our findings revealed fatigue and depressive symptoms to be dominant in iMCD patients, suggesting the routine use of fatigue and PHQ-9 scales to evaluate depressive symptoms in clinical practice. Furthermore, our study provides more detailed insights into the burden of care experienced by family members and caregivers compared to the study by Mukherjee and colleagues [17]. The paired analysis of patient-caregiver dyads in our study precisely demonstrates how disease characteristics impact the burden of care.

Fatigue is predominant in iMCD patients throughout the whole treatment process. This symptom is associated with worse general health status, more depressive symptoms, and lower working ability. Cancer-related fatigue is described as a distressing, persistent, subjective sense of physical, emotional, and/or cognitive tiredness or exhaustion related to cancer or cancer treatment that is not proportional to recent activity and interferes with usual functioning [36]. Proposed mechanisms for cancer-related fatigue include pro-inflammatory cytokines [37], hypothalamic–pituitary–adrenal (HPA) axis dysregulation [38], and circadian rhythm desynchronization[39]. These mechanisms may be interdependent, with the inflammatory state being a central component [40, 41]. Inflammatory symptoms and elevated serum levels of IL-6, a pro-inflammatory cytokine, are predominant in iMCD patients. A study has found a significant correlation between serum level of IL-6, serum cortisol and tumor-related symptoms,[42] which may explain the predominant fatigue symptom presented in iMCD patient. Screening and evaluating fatigue in cancer patients generally rely on PROM [36, 43–45], as fatigue is a subjective symptom. Similar to previous studies of other cancer types, our study found persistent fatigue in iMCD patients by PROMs, which adversely affects patients’ quality of life and working ability. Long-term fatigue during post-treatment in cancer have been reported to be related to the persistent activation of the immune system, prolonged inflammatory state and late effects of treatment on major organ systems [46, 47]. Since iMCD patients receive prolonged and continuous systemic therapy [4–6], longitudinal studies that evaluate fatigue before, during, and after iMCD treatment are needed.

Our findings underscore the prevalence of depression in iMCD patients, highlighting the need for increased attention to this significant comorbidity. We observed that iMCD patients currently receiving treatment demonstrated significantly higher levels of depression compared to untreated patients. This finding may be attributed to the cross-sectional nature of our study, as untreated patients might have relatively mild symptoms that do not necessitate treatment (Fig. 3A). Additionally, treatment-related factors such as side effects, economic burden, and caregiving burden could contribute to the depressive symptoms experienced by patients receiving treatment. Longitudinal studies are crucial for monitoring changes in depressive symptoms over time, particularly in relation to treatment status. Depression has been reported in POEMS syndrome with or without Castleman disease, with 38% of the patients reported major depression [48]. There has also been a high prevalence of depression reported in non-psychiatric inflammatory disorders [49] and in cancer [50]. The underlying pathophysiology of depression has been postulated to be linked to the persistent inflammatory state during diagnosis and treatment [51]. Inflammatory markers, such as IL-6, have been reported to be associated with an increased risk of depressive symptoms [52]. Given that iMCD is an inflammatory cancer characterized by increased serum IL-6 levels, our results are compatible with prior studies and implicated inflammation in the pathophysiology of depression in iMCD. Previous studies have also found that IL-6 neutralizing antibodies, such as Siltuximab, can improve depressive symptoms and PRO results in MCD patients [11, 53]. With screening tools such as the PHQ-9, depressive symptoms might be considered as a measurable outcome for IL-6 targeted treatment response in iMCD patients. In future studies of iMCD, more attention is needed in the timely screening, periodically evaluation, and precise intervention of depressive symptoms.

The result of CRO results suggested that the burden of caregiving for iMCD patients was mild but still had an impact on the general health of caregivers. Family caregivers play a vital role in providing support for cancer patients and essential resources for families affected by cancer [54]. The diagnosis and treatment of cancer have significant physical and psychological impacts on both patients and their caregivers [55]. In China, the decision-making model differs from that in Western countries, with a doctor-family-patient model based on Confucian philosophy being commonly observed [56]. Family caregivers bear the burden of care along with the patients and often have a key role in the medical decision-making process.

In our results, we found that self-criticism and feelings of lacking family support were prominent among caregivers of iMCD patients. As the burden of caregiving is known to be associated with caregivers’ mental health in cancer patients [57], it is crucial to periodically evaluate the caregiving burden and develop interventions to alleviate it. Our results indicated that the caregiving burden was associated with worse physical and mental health among iMCD caregivers in China, which is consistent with findings from previous studies. Additionally, our results showed that older and male caregivers exhibited more adverse effects on their health and social lives. Therefore, family-centered positive psychological interventions may be considered to improve the psychological health and quality of life for both iMCD patients and their caregivers.

Limitations of our study include the lack of longitudinal investigation into the changes of PRO. A longitudinal study observing the changes in PRO in the same patients over time would provide more compelling evidence and a more comprehensive understanding of the correlations between PRO and clinical treatment responses. Further research is necessary to explore the role of PRO in disease status and integrate PROMs in the evaluation of treatment responses. In our study, we utilized existing measurements for PROM and CROM that have been validated in other diseases among the Chinese population. Although these measurements are convenient and reliable for clinical use, they were not specifically tailored for iMCD patients and caregivers. While convenient and reliable for clinical use, the MCD-SS, designed for MCD patients including idiopathic and other subtypes, may not fully capture the unique symptom burden of iMCD. A specific symptom burden measurement for iMCD, validated in future studies, could provide a more reliable assessment of the full range of iMCD symptoms. Additionally, the reliance on the MCD-SS and its recall period of the previous 24 h may not adequately capture all symptoms. There is a need for appropriate assessments of general health for iMCD patients and caregiving burden for iMCD caregivers. Given the prominent presence of fatigue and depressive symptoms in iMCD patients, and their correlations with the burden of care, the development of disease-specific PROMs and CROMs for iMCD should be considered in future studies.

## Conclusion

Our study is one of the first studies to comprehensively assess the clinical symptom burden, quality of life, mental and psychological state, and social function of iMCD patients and caregivers. Our findings emphasize the need for increased attention to the management of fatigue and depressive symptoms in iMCD patients, and the caregiving burden, as evidenced by feelings of guilt and lack of family support. Longitudinal studies are needed to assess the correlations between PROMs and the clinical response to treatment in the future.

## Supplementary Information

Additional file1

## Data Availability

Data supporting the findings of this study are available within the paper and its Supplementary Information. Additional information are available from the corresponding author upon reasonable request.
