# Supplementary material for: Quality of life after idiopathic multicentric Castleman disease in China: a cross-sectional, multi-center survey of patient reported outcome and caregiver reported outcome
Source: Orphanet J Rare Dis. 2024 Dec 19;19:469. doi: 10.1186/s13023-024-03450-0 (PMC11660598; doi:10.1186/s13023-024-03450-0)
Supplement: Supplementary file 1 — Additional file1 [file 13023_2024_3450_MOESM1_ESM.pdf]

Supplementary Information

Table S1. Absenteeism, presenteeism, overall work impairment and activity impairment in iMCD patients measured by WPAI:GH.

WPAI:GH: Work Productivity and Activity Impairment Questionnaire: General Health.

Variables	n	Mean (SD)
Patients working for pay	84	
Work time missed due to health %	84	12·6 (25·0)
Work time missed due to health (those with missed time >0) %	37	28·5 (31·2)
Patients who actually worked in the past seven days	81	
Percent impairment while working due to health %	81	21·2 (21·8)
Percent impairment while working due to health (those with % impairment while working >0) %	57	31·3 (19·6)
Percent overall work impairment due to health	84	28·6 (29·1)
All patients	178	
Percent activity impairment due to health %	178	29·4 (24·8)
Percent activity impairment due to health (those with % activity impairment >0) %	141	37·1 (22·1)

Table S2. Demographic characteristics of iMCD patient-caregiver dyad.

Characteristics of iMCD patients	Patients-caregiver dyad (N=42)	All patients (N=178)	P value
Age, median (range)	42 (19-73)	43 (18-73)	0·430
Gender, N (%)			0·471
Male	25 (59·5%)	95 (53·3%)	
Female	17 (40·5%)	83 (46·6%)	
Sub type, N(%)			0·026
iMCD-TAFRO	7 (16·7%)	11 (6·2%)	
iMCD-NOS	35 (83·3%)	167 (93·8%)	
Duration from diagnosis, months	6·5 (1-100)	16 (1-280)	0·100
Treatment, N (%)			0·204
untreated	7 (16·7%)	29 (16·3%)	
in first-line	27 (64·3%)	95 (53·4%)	
≥ second-line	8 (19·0%)	38 (21·3%)	
in remission	0 (0%)	16 (9·0%)	
Education, N (%)			0·834
Primary education	2 (4·8%)	13 (7·3%)	
Lower secondary education	9 (21·4%)	35 (19·7%)	
Upper secondary education	12 (28·6%)	45 (25·2%)	
Advanced education	19 (45·2%)	81 (45·5%)	
Unkown	0 (0%)	4 (2·2%)	
**Characteristics of iMCD caregivers**	**Patients-caregiver dyad (N=42)**	**All caregivers (N=82)**	**P value**
Age, median (range)	45 (25-73)	42 (21-73)	0·990
Gender, N (%)			0·535
Male	14 (33·3%)	32 (39·0%)	
Female	28 (66·7%)	50 (61·0%)	
Relationship, N (%)			0·605
Spouse	25 (59·5%)	39 (47·6%)	
Parents	9 (19·0%)	19 (23·2%)	
Children	7 (16·7%)	21 (25·6%)	
Siblings	2 (4·8%)	3 (3·7%)	
Education, N (%)			0·703
Primary education	0 (0%)	2 (2·4%)	
Lower secondary education	9 (21·4%)	18 (22·0%)	
Upper secondary education	10 (23·8%)	19 (23·2%)	
Advanced education	23 (54·8%)	41 (50·0%)	
Unkown	0 (0%)	2 (2·4%)	
Past medical history, N (%)			0·825
Healthy	34 (81·0%)	65 (79·3%)	
*Unhealthy	8 (19·0%)	17 (20·7%)	
Duration of caregiving, N (%)			0·692
Less than 3 months	11 (26·2%)	18 (22·0%)	
3-6 months	6 (14·3%)	13 (15·9%)	
7-12 months	4 (9·5%)	4 (4·9%)	
More than 1 year	21 (50·0%)	47 (57·3%)	
Frequency of caregiving, N (%)			0·928
Everyday	24 (57·1%)	45 (54·9%)	
More than 4 days a week	3 (7·1%)	6 (7·3%)	
1-3 days a week	2 (4·8%)	8 (9·8%)	
Several days a month	6 (14·3%)	7 (8·5%)	
Several days a year	4(9·5%)	9 (11·0%)	
Independent	1 (2·4%)	2 (2·4%)	
Prefer not to answer	2 (4·8%)	5 (6·1%)	

Supplementary Figure Legends

Figure S1. Place for medical treatment and place of residence of iMCD patients in China. (A) Place for medical treatment of 178 iMCD patients. (B) Place of residence of 178 iMCD patients.

Figure S2. Prevalence of absenteeism, presenteeism, overall work impairment and activity impairment in iMCD patients.

Figure S3. Internal correlations in (a) patient reported outcomes and (b) caregiver reported outcomes. Spearman's rank correlation coefficient, or Spearman rho, is annotated in the figure. Correlations with p value < 0·05 are displayed. [1]Gender: male=1, female=2; [2]Health status: healthy=1, unhealthy=2.

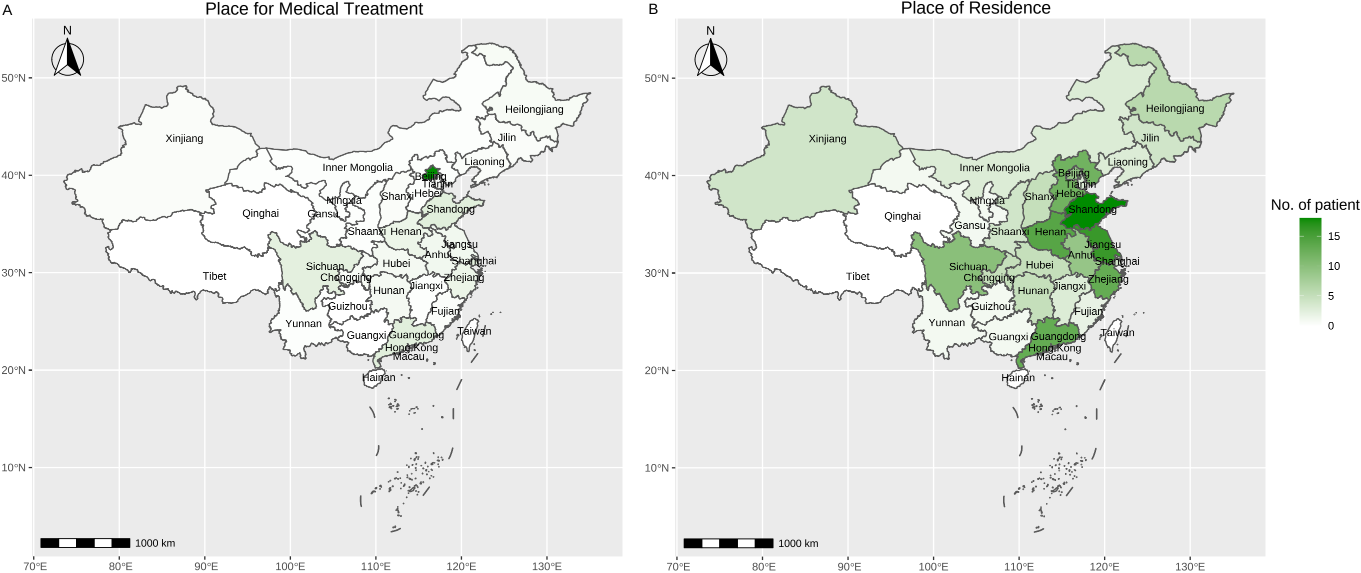
A
Place for Medical Treatment
B
Place of Residence
N
Xinjiang
Tibet
Qinghai
Gansu
Inner Mongolia
Ningxia
Shaanxi
Shanxi
Beijing
Tianjin
Hebei
Liaoning
Jilin
Heilongjiang
Shandong
Henan
Jiangsu
Anhui
Shanghai
Hubei
Sichuan
Chongqing
Hunan
Jiangxi
Zhejiang
Guizhou
Yunnan
Guangxi
Guangdong
Fujian
Taiwan
Hong Kong
Macau
Hainan
1000 km
50°N
40°N
30°N
20°N
10°N
70°E
80°E
90°E
100°E
110°E
120°E
130°E
No. of patient
15
10
5
0

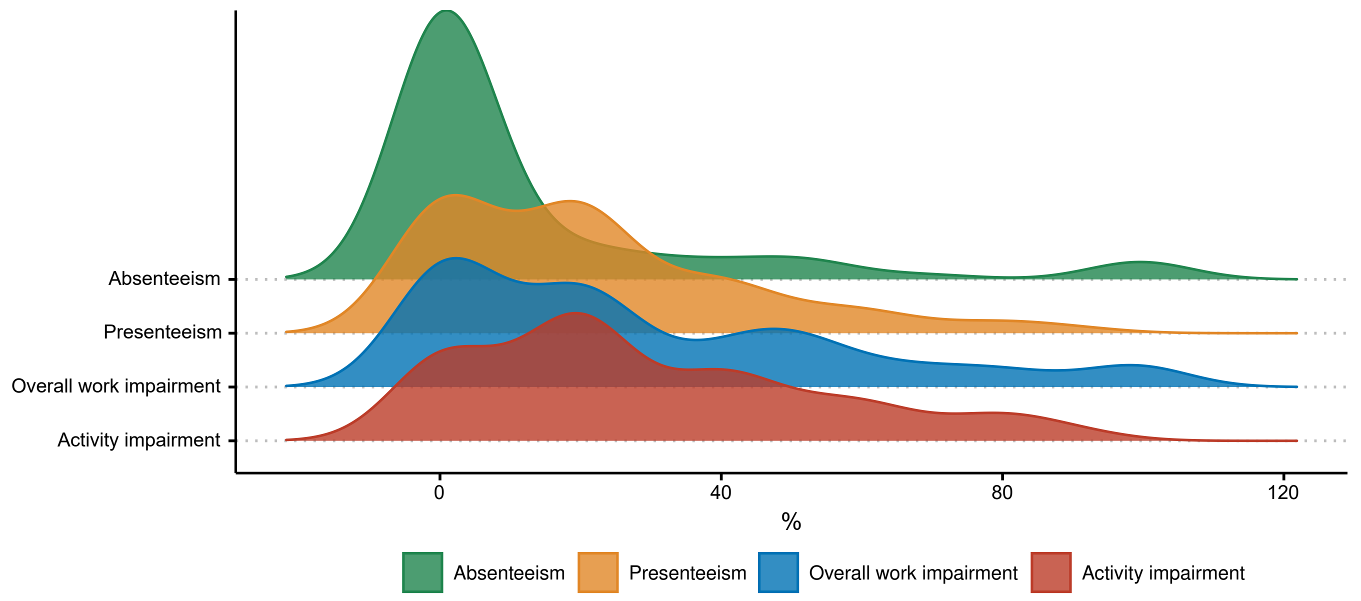

Absenteeism
Presenteeism
Overall work impairment
Activity impairment
0
40
80
120
%
Absenteeism
Presenteeism
Overall work impairment
Activity impairment

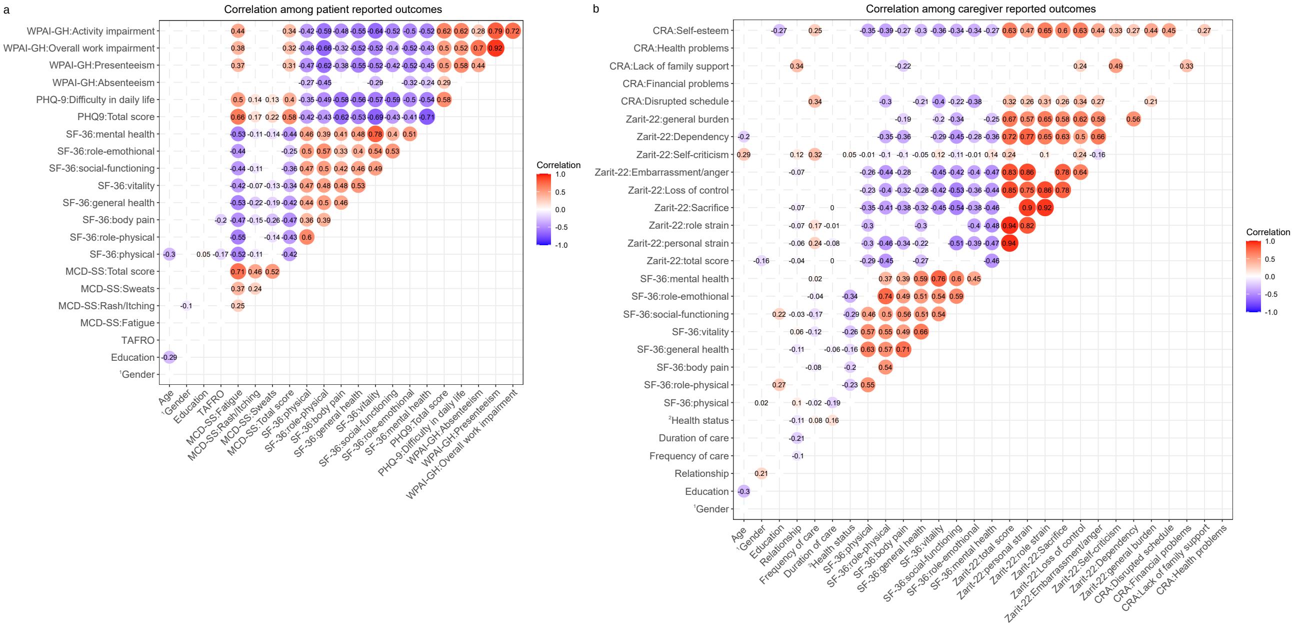
a
Correlation among patient reported outcomes
WPAI-GH:Activity impairment
WPAI-GH:Overall work impairment
WPAI-GH:Presenteeism
WPAI-GH:Absenteeism
PHQ-9:Difficulty in daily life
PHQ9:Total score
SF-36:mental health
SF-36:role-emothional
SF-36:social-functioning
SF-36:vitality
SF-36:general health
SF-36:body pain
SF-36:role-physical
SF-36:physical
MCD-SS:Total score
MCD-SS:Sweats
MCD-SS:Rash/Itching
MCD-SS:Fatigue
TAFRO
Education
1Gender
Age
Correlation
1.0
0.5
0.0
-0.5
-1.0
b
Correlation among caregiver reported outcomes
CRA:Self-esteem
CRA:Health problems
CRA:Lack of family support
CRA:Financial problems
CRA:Disrupted schedule
Zarit-22:general burden
Zarit-22:Dependency
Zarit-22:Self-criticism
Zarit-22:Embarrassment/anger
Zarit-22:Loss of control
Zarit-22:Sacrifice
Zarit-22:role strain
Zarit-22:personal strain
Zarit-22:total score
SF-36:mental health
SF-36:role-emothional
SF-36:social-functioning
SF-36:vitality
SF-36:general health
SF-36:body pain
SF-36:role-physical
SF-36:physical
2Health status
Duration of care
Frequency of care
Relationship
Education
1Gender
Age
Correlation
1.0
0.5
0.0
-0.5
-1.0
